# On the Treatment of Airline Travelers in Mathematical Models

**DOI:** 10.1371/journal.pone.0022151

**Published:** 2011-07-25

**Authors:** Michael A. Johansson, Neysarí Arana-Vizcarrondo, Brad J. Biggerstaff, J. Erin Staples, Nancy Gallagher, Nina Marano

**Affiliations:** 1 Division of Vector-Borne Diseases, Centers for Disease Control and Prevention, San Juan, Puerto Rico, United States of America; 2 Division of Vector-Borne Diseases, Centers for Disease Control and Prevention, Fort Collins, Colorado, United States of America; 3 Division of Global Migration and Quarantine, Centers for Disease Control and Prevention, Atlanta, Georgia, United States of America; Massey University, New Zealand

## Abstract

The global spread of infectious diseases is facilitated by the ability of infected humans to travel thousands of miles in short time spans, rapidly transporting pathogens to distant locations. Mathematical models of the actual and potential spread of specific pathogens can assist public health planning in the case of such an event. Models should generally be parsimonious, but must consider all potentially important components of the system to the greatest extent possible. We demonstrate and discuss important assumptions relative to the parameterization and structural treatment of airline travel in mathematical models. Among other findings, we show that the most common structural treatment of travelers leads to underestimation of the speed of spread and that connecting travel is critical to a realistic spread pattern. Models involving travelers can be improved significantly by relatively simple structural changes but also may require further attention to details of parameterization.

## Introduction

The global airline network has brought the entire world closer together than ever before, creating an environment in which pathogens can readily spread to distant locations. Influenza viruses are perhaps the most widely recognized example of this phenomena [1], [2], but by no means the only one [3], [4], [5]. Study of past spread and discussion about potential mitigation efforts has lead to a significant body of literature investigating the use of mathematical models to predict global pathogen spread and to assess the potential effectiveness of various interventions [3], [6], [7], [8], [9], [10], [11], [12], [13].

These models are generally framed as metapopulation models, with spatially discrete populations connected by transportation networks. The discrete populations themselves are subdivided in terms of infection status with compartments for susceptible, incubating, infectious, and recovered individuals. Development of these models presents many challenges related to the characterization of the populations, the disease, and relevant travel patterns. We focus here on oft-neglected assumptions related to the characterization of travel which may affect the speed and pattern of epidemic growth and spread.

**Figure 1:**
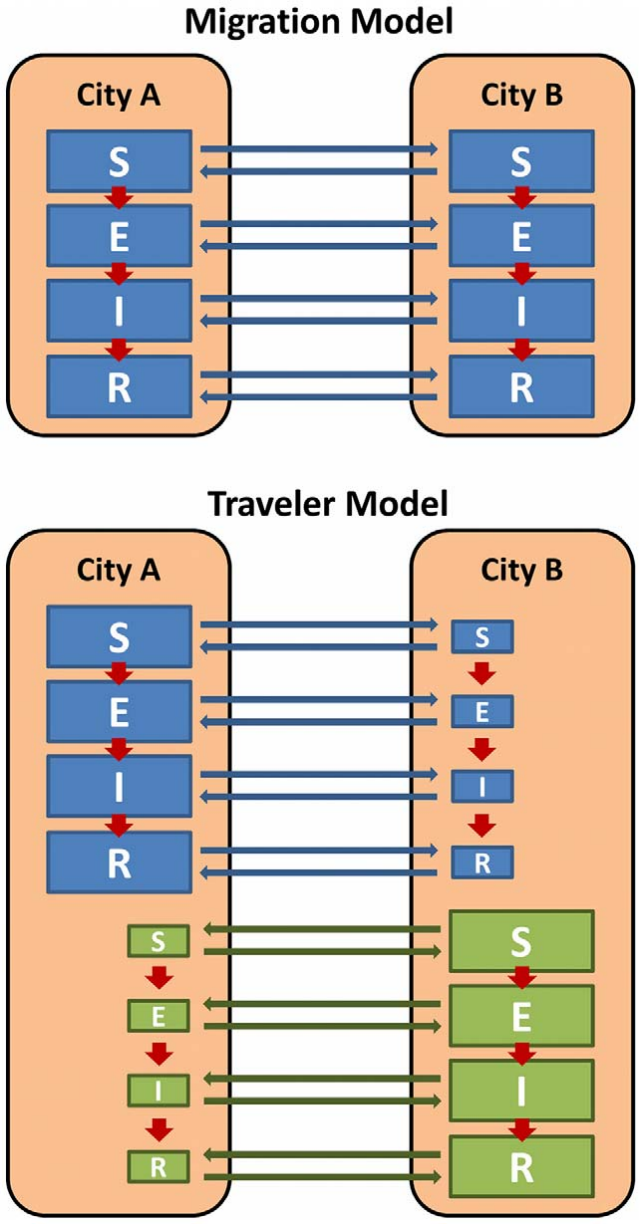
Alternative travel model structures. Two different model structures are shown using a simplified, two-city, Susceptible (S)-Exposed (E)-Infected (I)-Recovered (R) model. In the migration model, travelers represent migrants, mixing the populations of the two cities, City A and B. In the traveler model, some residents from City A (blue) and City B (green) travel temporarily to the other city, but eventually return to their original city. In each model, individuals may progress through the infection stages (red arrows), the rates of which may be city-dependent.

One fundamental assumption in many models is that travelers are, in essence, migrants; people who move permanently or semi-permanently from one geographic population to another [3], [6], [7], [8], [9], [10], [11], [12], [13]. In the real world, some travelers are migrating, but for most, travel is temporary. For example, each year there are approximately 175 million total admissions to the United States and approximately 1.1 million people obtain legal permanent resident status [14]. Thus, less than 1% of travelers entering the U.S. are migrating. An alternative to adopting this approach is to segregate travelers from local residents structurally, maintaining them as separate groups inhabiting the same area as a resident population (Figure 1). Separating travelers from residents this way allows them to return to their place of origin after a temporary stay in their destination.

It is also generally assumed that exposure occurs via mass action, i.e., that all susceptible individuals in a given location are exposed with the same probability to the infectious agent. Even without considering travel, however, there is significant stratification within populations and people do not all interact equally [15]. For travelers, exposure is often related to their purpose for travel. The majority of outbound US traveler purposes can be classified as leisure/recreation/holidays (40%), visiting friends and relatives (34%), or business (18%) [16]. Those visiting friends and relatives are more likely to stay in private homes, to stay longer, to interact more closely with the local population, and to have higher exposure risks [16], [17], [18], [19], [20], [21].

Infection with a pathogen may also have a direct impact on travel. Most models assume that infectious travelers are ill and thus are unlikely to travel [6], [7], [8], [9], [12]. However, infectious people are not necessarily ill and even if they are, they may travel anyway if they can.

Characterization of a travel network itself is another challenge. Many models quantify travel volumes or frequencies in terms of the number of seats available on direct flights between each location and average occupancy on those flights [6], [7], [8], [9], [10]. While this may accurately classify non-connecting travel (∼80% of travel itineraries based on U.S Department of Transportation sampled itinerary data), it completely misses travelers who fly on itineraries containing one or more connections. As a result, this simplification underestimates long-distance travel, because no one can fly between cities that are not directly connected, and overestimates short-distance travel, because it assumes that all travelers are traveling direct. Limiting travel in this way may lead to alteration and truncation of the pattern of spread, especially when transmission risk is heterogeneous across the network.

We used mathematical models of two generalized pathogens, one directly transmitted (modeled on severe acute respiratory syndrome-associated coronavirus (SARS-CoV)) and one vector-borne (modeled on dengue viruses (DENV)) to evaluate the effects of different structural model designs and characterizations of airline travel on the speed and pattern of epidemic spread. In addition to evaluating directly transmitted pathogens, we also include a vector-borne pathogen because the dependence on a vector increases the geographical heterogeneity in transmission dynamics and may alter the effects of travel-related assumptions for a pathogen.

## Methods

### Model description

Two base models were constructed, one for the directly transmitted pathogen and one for the vector-borne pathogen. Described in detail in Text S1, both models are stochastic metapopulation models with discrete daily time steps. Each model includes 141 cities selected to represent a realistic sample network with varied connectivity and population sizes. Travel and infection are assumed to be independent. Travel rates and stay durations were estimated using sampled travel data (Official Airline Guide (OAG), www.oagaviation.com/Solutions/AnalysisTools/Traffic/t100inet.html and US Department of Transportation, www.transtats.bts.gov/Tables.asp?DB_ID=125). Each city in the model had susceptible, incubating, infectious, and recovered/immune compartments, with separate compartments for residents and travelers. Though maintained separately, residents and travelers contribute equally to the local force of infection and are exposed to infection at the same rate. The incubating and infectious compartments all included sub-compartments to allow more realistic incorporation of the time periods spent in each [22]. For models of the directly transmitted pathogen, the rates of effective contact, time to progression to the infectious compartment, and time to recovery are based on previous studies [23], [24]. In contrast to previous efforts, our model has been simplified to specifically address the influence of travel in the absence of any interventions.

To introduce straightforward geographic variability in the models of the vector-borne pathogen, we randomly selected 10 cities to have *Aedes aegypti* DENV-vector mosquito populations. Each of these cities was seeded with two susceptible mosquitoes per human. Upon effective exposure to infectious humans, vectors moved through incubating sub-compartments before becoming infectious at rates estimated based on previous models [25]. Vectors from all compartments were susceptible to nominal mortality (defined globally based on [26]) and were replaced by new susceptible vectors at the same rate. Human infection is dependent on estimated effective contact rates [27], [28] and the prevalence of infectious vectors.

### Model alterations to assess specific assumptions

To test the effect of treating travelers as migrants rather than as temporary travelers, we compared the base, “traveler” models with “migration” models in which we eliminated the traveler compartments and assumed that all travelers immediately mixed with local populations upon arrival in a new city.

To test the role of heterogeneous travel patterns, we divided travelers into two compartments based on approximations of the duration of stay for those residing in hotels versus private homes. In the base models, travelers stay an average of 18 days in their destination (exponentially distributed) [16]. In the modified models, we assume that approximately 60% of travelers stay in hotels [16]. These travelers are also less likely to stay as long, so we modified the length of stay to average 14 days for those staying in hotels, and 24 days for those staying in homes. We therefore maintained the average length of stay of approximately 18 days used in the base models. We then prevented transmission associated with hotel or home stays alternatively by eliminating interactions for the group of interest so they could neither contribute to the force of infection nor be exposed.

To test the importance of infectious travelers, we compared the base models, which allow individuals to travel regardless of infection status, with models in which infectious individuals do not travel.

We also assessed three different travel network parameterizations: seat-based direct-travel only, connecting-travel-inclusive, and a skewed version of the connecting-travel. Connecting travel was estimated using a regression model of sampled itinerary data and covariates based on travel network characteristics (described in detail in Text S1). For the models with skewed travel, we used the same overall passenger flow as the connecting-travel-inclusive models but changed directionality so that 2/3 of travelers between tropical and non-tropical cities are residents of non-tropical cities, leaving 1/3 as residents of the tropical cities.

### Model comparison

To analyze the effect of different assumptions, we ran simulations of each model under the different sets of assumptions. In each simulation, it was assumed that the global population was 100% susceptible and 10 infectious individuals were introduced into a single city. The origin city was selected from the subset of cities containing vector populations and was used for simulated epidemics of both the directly transmitted and the vector-borne pathogens. The same city was used as the origin city for the directly transmitted model.

Different models were compared by performing 100 simulations of each model and comparing quantitative outcomes via regression analysis. The timing of the first autochthonous transmission event in each city and the final epidemic size in each city were compared using a linear Gaussian regression with city as a covariate to control for intra-city correlation across simulations. Intra-simulation correlation was not found, i.e., earlier or later introduction was related to the city and the model, but not to the simulation. Statistical analyses were performed in R version 2.11.1 [29].

## Results

### Temporary travel versus migration

**Figure 2:**
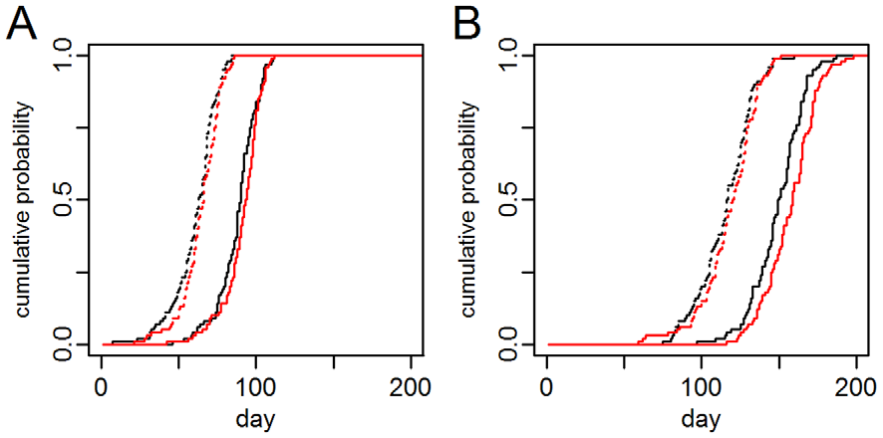
Timing of first autochthonous human infection: migration vs. traveler model. Each line is the empirical cumulative probability (for 100 simulations) of the first autochthonous transmission event in a single city in a single model. The dotted lines are for a city closely connected to the origin city for the traveler (black) and migration (red) models. The solid lines are for a more distal city. A. is the directly transmitted pathogen and B. is the vector-borne pathogen.

We contrasted two different model designs, one treating all travelers as migrants (migration models) and one treating them as temporary travelers (traveler models, the base models). For the directly transmitted pathogen, the pathogen introduction caused a pandemic. Figure 2a shows representative cumulative distributions of the time of the first autochthonous human infection in two cities. Controlling for the inter-city variation, the average onset of autochthonous transmission in the traveler model was 3.3 days earlier (95% CI: 3.0–3.6 days) than in the migration model. For the vector-borne pathogen, this difference was more pronounced (Figure 2b). In the nine cities with vector populations, introduction occurred an average of 6.7 days earlier (95% CI: 5.0–8.3 days) in the traveler model than the migration model.

**Figure 3:**
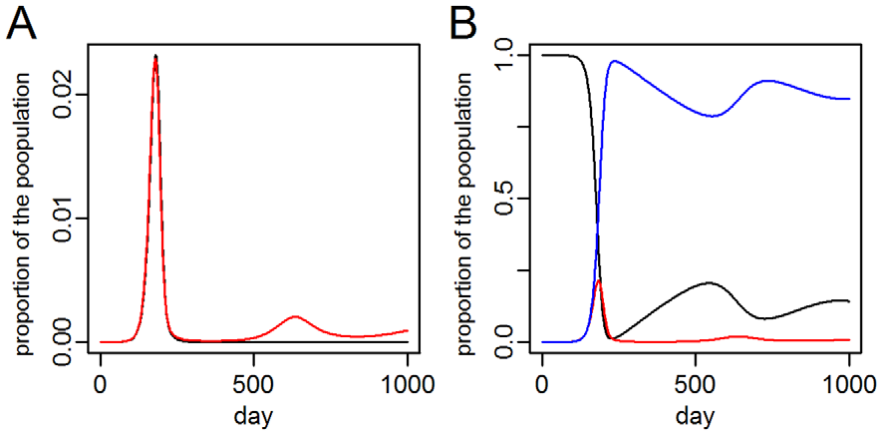
Epidemic recurrence in the city where the epidemic originates. A. The mean proportion of the population newly infected per day for the traveler (black) and migration (red) models. The pathogen only persists in the migration model and causes a second outbreak approximately two years after the first. B. The mean proportion of the population susceptible (black), incubating and infectious (red), and recovered (blue) in the migration model over time. Approximately 20% of the population is replaced by incoming susceptibles between the two epidemics.

The final epidemic size was slightly decreased in the migration model (0.079%, 95% CI: 0.088–0.069%) compared to the traveler model for the directly transmitted pathogen. However, long-term dynamics were generally consistent with the pandemic ending in global extinction of the pathogen within 1.5 years. In contrast, there were long-term differences in the models of the vector-borne pathogen. In the traveler model, initial epidemics were followed by global extinction of the pathogen within two years (Figure 3A). In contrast, the migration model allowed continued mixing of the population leading to susceptible replenishment in affected cities. Once the initial outbreak in the initial city subsided, 18.0% (95%CI: 17.6–18.3%) of that population was replaced by immigrating susceptibles within one year. Because of reintroduction of the pathogen or residual transmission from the previous epidemic, this replenishment led to recurrent outbreaks (Figure 3B). The continual migration also slowly homogenizes all model populations such that, after many years, a global equilibrium is reached. In comparison, the traveler model results in a stable, geographically heterogeneous distribution as soon as extinction of the pathogen occurs.

### Heterogeneity among travelers

We designed additional traveler models (heterogeneous-traveler models), which had different periods of stay for travelers staying in hotels compared to travelers staying in homes. We investigated the effects of the traveler heterogeneity in the model with the directly transmitted pathogen by blocking the interaction of travelers staying in hotels with the rest of the population (i.e. residents or travelers staying in homes). This delayed spread to other cities by an average of 9.2 days (95% CI: 8.9–9.5 days) and reduced ultimate epidemic size by 0.36% (95% CI: 0.34–0.37%). Blocking transmission associated only with travelers staying in homes delayed spread by 5.2 days (95% CI: 4.9–5.5 days) and decreased final epidemic size by 0.12% (95% CI: 0.11–0.13%).

For the heterogeneous-traveler model with the vector-borne pathogen, blocking the interaction of travelers staying in hotels with mosquitoes reduced the speed of spread by an average of 11 days (95% CI: 9–13 days) and decreased the ultimate size of the epidemics by 0.40% (95% CI: 0.39–0.41%). In contrast, blocking traveler-associated transmission in homes slowed spread by 5.5 days (95% CI: 3.8–7.3 days) and decreased final epidemic size by 0.36% (95% CI: 0.35–0.37%).

### Infectious travelers

In the base model, infectious individuals travel with the same frequency as any other individual. We constructed additional models (no-infectious-travel models), in which infectious individuals did not travel, i.e., infectious individuals neither leave their city of residence nor do they return home from another city until they have recovered. Eliminating infectious travel resulted in spread to other locations occurring approximately 3.8 days (95% CI: 2.6–5.0 days) and 3.9 days (95% CI: 2.2–5.5 days) later in the models with directly transmitted and vector-borne pathogens, respectively. In the no-infectious-travel models, the final epidemic size was slightly decreased (mean difference: −0.018%, 95% CI: −0.028–−0.007%) for the directly transmitted pathogen, and was unchanged (mean difference: 0.0007%, 95% CI: −0.0006–0.0020%) for the vector-borne pathogen.

### Travel network characterization

**Figure 4:**
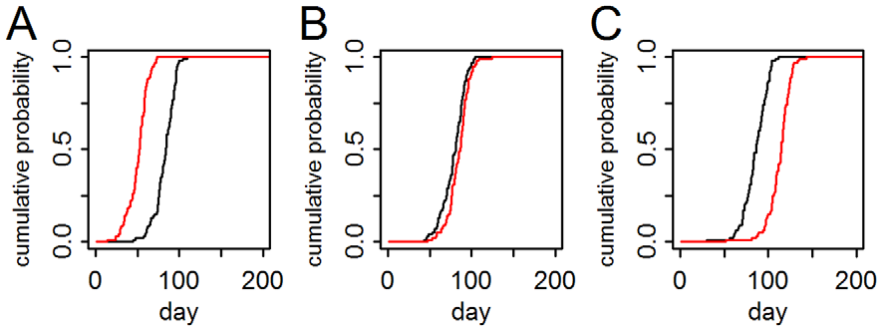
Timing of first autochthonous human infection: direct- vs. connecting-travel network model with the directly transmitted pathogen. Each line is the empirical cumulative probability (for 100 simulations) of the first autochthonous transmission for the connecting-travel model (black) and direct-travel model (red) for 3 cities: A. directly connected to the origin city; B. non-directly connected, intermediate distance city; and C. a distant city.

We first assessed the role of different assumptions regarding the structure of the airline travel network using the base models (with connecting travel) compared to models with travel restricted to single-leg, direct flights (direct-travel-only models). Overall, for the directly transmitted pathogen, the onset of autochthonous transmission in secondary cities in the direct-travel model occurred on average 10.5 days (95% CI: 10.1–11.0 days) later (Figure 4). Although spread was generally slower, this was not universally the case. For 42 (30%) of the 141 cities, spread was faster (Figure 4A), for 90 cities (64%), slower (Figures 4B and 4C), and for 9 cities (6%), there was no significant difference. Average epidemic size was unchanged (95% CI: −0.040–0.008%).

**Figure 5:**
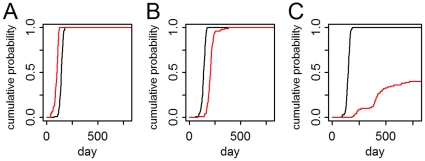
Timing of first autochthonous human infection: direct- vs. connecting-travel network model with the vector-borne pathogen. Each line is the empirical cumulative probability (for 100 simulations) of the first autochthonous transmission for the connecting-travel model (black) and direct-travel model (red) for 3 cities: A. directly connected to the origin city; B. non-directly connected, intermediate distance city; and C. a distant city.

Models of the vector-borne pathogen had significant geographical heterogeneity and showed more drastic effects when travel characteristics were modified (Figure 5). Under the direct-travel-only model, spread to the nine cities with vector populations was delayed by an average of 188 days (95% CI: 181–196 days). However, there was significant variation between cities with average introduction occurring as early as 48 days (95% CI: 33–62 days) earlier for the only directly connected city (Figure 5A), to 330 days (95% CI: 317–345 days) later for a distant city. For one city, introduction was sporadic, occurring in only 40 of the 100 simulations (Figure 5C). Compared to the connecting-travel model, the epidemics in the direct-travel model affected 1.9% (95% CI: 1.6–2.1%) more people.

The connecting-travel network was then modified to skew traffic such that more of the travelers would be residents of non-tropical areas visiting tropical areas than vice versa. Spread occurred approximately 1.6 days (95% CI: 1.3–1.9 days) earlier in the skewed-travel model for the directly transmitted pathogen. In non-tropical cities, epidemic size did not change (95% CI: −0.006–0.015%), but in tropical cities, average epidemic size increased by 0.57% (95% CI: 0.52–0.62%). The skewed-travel model of the vector-borne pathogen behaved similarly with a non-significant decrease in the speed of spread (95% CI: −0.3–3.0 days). Epidemic size decreased in the non-tropical cities by 0.040% (95% CI: 0.028–0.052%) and increased in the tropical cities by 0.26% (0.23–0.29%).

## Discussion

The global connectivity of air travel presents a significant public health issue as infected travelers may move pathogens long distances in short time periods. We explored various assumptions and considerations related to the incorporation of travel in models, including the structural treatment of travelers, the role of infectious travelers, the interaction of travelers with local communities, and the characterization of the travel network. Analyzed outcomes included both local epidemic characteristics (epidemic size) and global characteristics (the speed of spread).

### Model outcomes

Most of the assumptions we tested resulted in relatively small modifications to local transmission, affecting the size of epidemics by less than one percent. For example, introducing heterogeneity among travelers reduced the average epidemic size in all models by a maximum of approximately 0.40%. These reductions occur because a proportion of travelers are never exposed, thus reducing the effective susceptible population size. A greater change in local epidemic size was seen in the direct-travel-only model for the vector-borne pathogen. The direct-travel-only model assumes that every seat is potentially used by a direct traveler as opposed to a seat potentially used by a connecting traveler. This overestimation of direct travel increases the overall volume of travel and thus leads to greater local replenishment of susceptibles, a phenomenon which takes on added importance when transmission is spatially heterogeneous. Though the mechanism is different, the effect of susceptible replenishment is also seen in the migration model. In this case, the replenishment of approximately 18% of the population within a single year led to recurrent epidemics, something completely absent from the traveler model.

Another critical outcome of interest from these models is the speed of spread across the network. Treating travelers as migrants reduced the speed of spread to other areas by approximately 3.3 and 6.7 days in models of the directly transmitted and vector-borne pathogens, respectively. This difference is likely due to proportional changes in the travelling population. Once an epidemic begins in a given city, travelers leaving that city will contain a mix of previously exposed (i.e. immune) individuals proportional to the general population. In the traveler model, this proportion is reduced by half because only half of the travelers are outgoing residents; the other half are visitors returning to their home. Because there are more susceptible travelers leaving and returning to cities where the pandemic has yet to arrive, the likelihood of a newly exposed individual arriving is slightly increased in the traveler model.

Preventing infectious individuals from travelling also led to delayed introduction by an average of 3.8 and 3.9 days for the models of the directly transmitted and vector-borne pathogens, respectively. Adding heterogeneity to exposure among travelers eliminates some potential infections among travelers and thus has the same effect.

Using different travel-network designs had an even greater effect on spread. Assuming that no travelers travel beyond the cities directly connected to their residential city delayed spread of the directly transmitted pathogen by an average of 10.5 days and the vector-borne pathogen by an average of 188 days. For both, that effect was spatially heterogeneous. The cities with high connectivity to the origin city experienced early introduction and distant cities, delayed introduction. For the vector-borne pathogen, the effects are more evident because cities with vector populations are not necessarily directly connected to each other. Of the ten randomly selected cities with vectors, a single city was directly connected to the origin city and four cities were not directly connected to any other city with a vector population. For these isolated cities, the pathogen could only arrive via an individual who is exposed in one city, returns to his/her vector-free home city and later travels to another city before recovering. With the large, intervention-free epidemics produced in our model, spread to these isolated cities did reliably occur except for in the city where an epidemic only occurred in 40% of the simulations (Figure 5C). With smaller epidemics this truncation of spread would likely be more common.

Skewing the flow of travelers to increase the proportion of travelers visiting tropical areas from non-tropical areas resulted in a slightly increased speed of spread in models of the directly transmitted pathogen (approximately 1.6 days). This likely resulted from the fact that the origin city was a non-tropical city, and thus infected residents were more likely to leave. For the model of the vector-borne pathogen, skewed travel did not significantly impact the speed of spread.

### General considerations

The models described here present various scenarios based on different assumption sets for two pathogens with particular transmission parameterizations. For example, the ratio of the incubation period to the amount of time spent in other areas is an important determinant of spread dynamics [30]. When incubation periods are much shorter than the amount of time spent in other areas, individuals are less likely to be incubating or infectious when they return home. For the models discussed here, the average durations of infection in humans were 11.22 and 9.5 days for SARS-CoV and DENV, respectively (including the incubating and infectious stages as defined here). In this case, the duration of infection is less than the average duration of stay (18 days), so some travelling individuals will recover before returning home. Thus, with shorter durations of infection or longer stays, the rate of spread will decrease, and with longer durations of infection and shorter stay, it will increase.

Though our results are particular to the systems we have analyzed, they demonstrate important generalities. The assumptions analyzed are related to both model structure and parameterization. The traveler model, for example, is essentially a structural upgrade relative to the migration model as it requires a single new parameter: the duration of stay. Though we used a universal exponential distribution to describe the duration of stay in the base model, it could also be parameterized as location-specific or time-dependent. The delayed introduction and the long-term persistence associated exclusively with the unrealistic, yet mostly commonly used, migration-style model suggest that it is not ideally suited to these types of studies. Developing a traveler model rather than a migration model requires a single additional parameter and a multiplicative increase in structural complexity as each location-compartment needs to be replicated in each other location. This increase in structural complexity, however, is not a formidable barrier given the power of modern day computers.

Travel for infectious individuals is both a structural and parameterization issue. For pathogens causing particularly severe disease, it may be completely realistic for no infectious individuals to travel, but for pathogens associated with mild disease or asymptomatic infection, infectious individuals may travel at the same rate as any other individual. However, data on differential travel practices for infectious individuals is rare. In the absence of solid data, we favor having infectious individuals travel at the same rate as others, thus avoiding the delays in spread induced by an unsubstantiated assumption.

Segregating exposure in the heterogeneous-traveler model for travelers staying in homes versus hotels also reduced the speed of spread. If adequate data exist to resolve different groups and different risk factors, it is advisable to include them in the model. The necessary structural changes are relatively straightforward; the challenge lies in the parameterization.

The final assumption we assessed was the parameterization of the travel network. It had the greatest effect on the results. Assuming that travelers only travel between cities connected by direct flights biased the speed of spread in both models and resulted in truncation of some of the vector-borne pandemics. As discussed above, truncation may result from spatial heterogeneity in pathogen transmission. Spatial heterogeneity may be somewhat overstated in our vector-borne model, but some level of spatiotemporal heterogeneity is generally present even for directly transmitted pathogens [31], [32]. Assuming that the flow of travelers between two locations is equivalent in each direction also has implications on the spread, making early spread to some locations more likely than to others.

The greatest hurdle for developing more realistic travel networks for models is the difficulty of obtaining adequate data. As discussed in Text S1, we took an approach with some similarities to that of Epstein et al. [12], characterizing connecting travel based on relatively accessible US sample itinerary and global air travel network data. Lessler et al. [33] also used the US sampled data to compare air travel network models with the goal of reducing their complexity. While their proposed, simplified model replicates actual data well, it is not easily extendable to a global scale where there are less input data and is not conducive to the explicit treatment of travelers that we have described. Accurate characterization of global air travel requires a substantial sample of global itineraries with accompanying data to help determine the duration of stay and likely exposure risks such as staying in hotels versus homes. It is critical to know where people truly travel (actual origin and destination), how long they stay in their destination, and how travel patterns vary throughout the year. Better data on true origin and destination would also help to classify the directional flow of travelers such as in the skewed-travel model presented here. Although the data requirements for full parameterization of travel are extensive, the necessary data exist, but they are proprietary and the cost of their acquisition is prohibitive for most scientific studies.

### Conclusions

The effects discussed here may be more or less pronounced for other pathogens. Notably, the importance of travel network assumptions is particularly important in the presence of geographic heterogeneity. Based on our findings, both the structural treatment of travelers and the consideration of connecting travel may be more important than currently recognized.

In future modeling efforts, we suggest at minimum assessing the sensitivity to the structural changes considered here, in particular those related to infectious travelers and treating travelers as temporary visitors rather than migrants. Improving some of the other deficiencies discussed here is also important but will require more extensive efforts due to the complexity of the problems and the paucity of relevant data.

## Supporting Information

Text S1 **Detailed description of the models and parameters used.**
(DOCX)

## References

[1] Grais RF, Ellis JH, Glass GE (2003). Assessing the impact of airline travel on the geographic spread of pandemic influenza.. European Journal of Epidemiology.

[2] Fraser C, Donnelly CA, Cauchemez S, Hanage WP, Van Kerkhove MD (2009). Pandemic potential of a strain of influenza A (H1N1): early findings.. Science.

[3] Hufnagel L, Brockmann D, Geisel T (2004). Forecast and control of epidemics in a globalized world.. Proceedings of the National Academy of Sciences of the United States of America.

[4] Wilder-Smith A, Gubler DJ (2008). Geographic expansion of dengue: the impact of international travel.. Medical Clinics of North America.

[5] Soumahoro MK, Fontenille D, Turbelin C, Pelat C, Boyd A (2010). Imported chikungunya virus infection.. Emerging Infectious Diseases.

[6] Rvachev LA, Longini IM (1985). A Mathematical-Model for the Global Spread of Influenza.. Mathematical Biosciences.

[7] Grais RF, Ellis JH, Kress A, Glass GE (2004). Modeling the spread of annual influenza epidemics in the U.S.: the potential role of air travel.. Health Care Manag Sci.

[8] Flahault A, Vergu E, Coudeville L, Grais RF (2006). Strategies for containing a global influenza pandemic.. Vaccine.

[9] Cooper BS, Pitman RJ, Edmunds WJ, Gay NJ (2006). Delaying the international spread of pandemic influenza.. PLoS Medicine.

[10] Colizza V, Barrat A, Barthelemy M, Valleron AJ, Vespignani A (2007). Modeling the worldwide spread of pandemic influenza: baseline case and containment interventions.. PLoS Medicine.

[11] Hollingsworth TD, Ferguson NM, Anderson RM (2007). Frequent travelers and rate of spread of epidemics.. Emerging Infectious Diseases.

[12] Epstein JM, Goedecke DM, Yu F, Morris RJ, Wagener DK (2007). Controlling pandemic flu: the value of international air travel restrictions.. PLoS One.

[13] Colizza V, Barrat A, Barthelemy M, Vespignani A (2007). Predictability and epidemic pathways in global outbreaks of infectious diseases: the SARS case study.. BMC Med.

[14] U.S. Department of Homeland Security (2009). Yearbook of Immigration Statistics: 2008..

[15] Mossong J, Hens N, Jit M, Beutels P, Auranen K (2008). Social contacts and mixing patterns relevant to the spread of infectious diseases.. PLoS Medicine.

[16] U.S. Department of Commerce - International Trade Administration (2009). Profile of U.S. Resident Travelers Visiting Overseas Destinations: 2008 Outbound..

[17] Ackers ML, Puhr ND, Tauxe RV, Mintz ED (2000). Laboratory-based surveillance of Salmonella serotype Typhi infections in the United States: antimicrobial resistance on the rise.. JAMA.

[18] Steinberg EB, Bishop R, Haber P, Dempsey AF, Hoekstra RM (2004). Typhoid fever in travelers: who should be targeted for prevention?. Clinical Infectious Diseases.

[19] Leder K, Tong S, Weld L, Kain KC, Wilder-Smith A (2006). Illness in travelers visiting friends and relatives: a review of the GeoSentinel Surveillance Network.. Clinical Infectious Diseases.

[20] Skarbinski J, James EM, Causer LM, Barber AM, Mali S (2006). Malaria surveillance–United States, 2004.. MMWR Surveillance Summaries.

[21] Fenner L, Weber R, Steffen R, Schlagenhauf P (2007). Imported infectious disease and purpose of travel, Switzerland.. Emerging Infectious Diseases.

[22] Wearing HJ, Rohani P, Keeling MJ (2005). Appropriate models for the management of infectious diseases.. PLoS Medicine.

[23] Donnelly CA, Ghani AC, Leung GM, Hedley AJ, Fraser C (2003). Epidemiological determinants of spread of causal agent of severe acute respiratory syndrome in Hong Kong.. Lancet.

[24] Riley S, Fraser C, Donnelly CA, Ghani AC, Abu-Raddad LJ (2003). Transmission dynamics of the etiological agent of SARS in Hong Kong: impact of public health interventions.. Science.

[25] Nishiura H, Halstead SB (2007). Natural history of dengue virus (DENV)-1 and DENV-4 infections: Reanalysis of classic studies.. Journal of Infectious Diseases.

[26] Focks DA, Haile DG, Daniels E, Mount GA (1993). Dynamic life table model for Aedes aegypti (Diptera: Culicidae): analysis of the literature and model development.. J Med Entomol.

[27] Scott TW, Amerasinghe PH, Morrison AC, Lorenz LH, Clark GG (2000). Longitudinal studies of Aedes aegypti (Diptera: Culicidae) in Thailand and Puerto Rico: blood feeding frequency.. Journal of Medical Entomology.

[28] Failloux AB, Vazeille M, Rodhain F (2002). Geographic genetic variation in populations of the dengue virus vector Aedes aegypti.. Journal of Molecular Evolution.

[29] R Development Core Team (2010). R: A Language and Environment for Statistical Computing..

[30] Keeling MJ, Rohani P (2002). Estimating spatial coupling in epidemiological systems: a mechanistic approach.. Ecology Letters.

[31] Alonso WJ, Viboud C, Simonsen L, Hirano EW, Daufenbach LZ (2007). Seasonality of influenza in Brazil: a traveling wave from the Amazon to the subtropics.. American Journal of Epidemiology.

[32] Shaman J, Pitzer VE, Viboud C, Grenfell BT, Lipsitch M (2010). Absolute humidity and the seasonal onset of influenza in the continental United States.. PLoS Biol.

[33] Lessler J, Kaufman JH, Ford DA, Douglas JV (2009). The cost of simplifying air travel when modeling disease spread.. PLoS One.

