# Supplementary material for: On the Treatment of Airline Travelers in Mathematical Models
Source: PLoS One. 2011 Jul 25;6(7):e22151. doi: 10.1371/journal.pone.0022151 (PMC3143116; doi:10.1371/journal.pone.0022151)
Supplement: Text S1 — Detailed description of the models and parameters used. (DOCX) [file pone.0022151.s001.docx]

**1. Model Structure**

**1.1 Overview**

**1.2 Travel**

**1.3 Directly-transmitted model**

**1.4 Vector-borne model**

**2. Parameterization**

**2.1 Populations**

**2.2 Travel**

**2.4 Infection dynamics**

**2.5 Transmissibility**

**3. References**

**1. Model Structure**

**1.1 Overview.** Here we describe the base models. Alterations to address different assumptions are described in the Methods section of the manuscript.

For both the directly-transmitted and vector-borne models, we separate each city into 2 distinct populations: travelers from other cities, *T*, and long-term residents of the city, *R*. Travelers are subdivided into compartments by their city of residence (so they may return there) and by infection state. Residents are subdivided only by infection state. For each daily time-step, we first calculate the number of travelers traveling between each city in each infection state and update each compartment accordingly. Next, we calculate changes in the infection state for each compartment within each city before updating those changes.

Lastly, we address population dynamics. For humans, we assume that there is no natural human mortality. Although this is clearly not realistic, human longevity greatly exceeds the complete course of an infection with either of the pathogens modeled here, and may thus be effectively ignored. We do address vector mortality, though, as vector mortality may play an important role in limiting transmission.

**1.2 Travel.** The first step is to calculate the number of travelers who move between each city pair. We assume that half of the outgoing travelers, *Y,* between locations *i* and *j* will be outgoing residents (and the other half, travelers from *j* returning to *j*). The probability of outgoing resident travel, *p_i,j_*, is thus half the number of expected outgoing travelers divided by the total resident population, *R* (including each compartment, *c*) currently in *i*:

$p_{i,j}=(Y_{i,j}/2)/(\sum_{c} R_{i,c})$.

For each resident compartment, *R_i,c_*, the number of outgoing travelers, Ω*^R^*, to each other city is sampled from a multinomial distribution:

$\Omega_{i,.,c}^{R}=\mathrm{Multinomial}(R_{i,c},p_{i,.})$.

For travelers who are residents of *j* but currently in *i*, the number returning to *j* in a time step is sampled from a binomial distribution for each traveler compartment, *T_i,j,c_*, with a constant probability of return, *ρ*:

.

Each resident compartment is then updated by adding the compartment-specific total number of returning travelers from each other city less the number of outgoing travelers:

$R_{i,c}\Leftarrow R_{i,c}+\sum_{j\neq i}^{N} (\Omega_{i,j,c}^{T}-\Omega_{i,j,c}^{R})$.

Each traveler compartment is also specific to the city of origin, so the change in one time step is the number of incoming compartment-specific travelers from the other city minus the number returning to that city:

$T_{i,j,c}\Leftarrow T_{i,j,c}+\Omega_{i,j,c}^{R}-\Omega_{i,j,c}^{T}$.

**1.3 Directly-transmitted model.** Humans are classified into four principal states relative to infection: susceptible, exposed/incubating, infectious, and recovered/immune (S, E, I, and R, respectively). Other models of SARS-CoV include further segregation regarding hospitalization and death [1,2]. We have omitted these for the sake of simplicity and because their numerical outcomes are not the outcomes of primary focus here. The adjusted parameterization is described in Section 2 below. The incubating and infectious periods are further subdivided to give more flexibility to the classification of these transient period [3].

All compartments are specific for city and city of origin (for the travelers), but all individuals in a given location (regardless of travel status) are exposed to the same conditions, so, for simplicity, we will refer to all human states without the city and travel status indices, e.g. *H_c_*, rather than *T_i,j,c_* and *R_i,c_*. Change components, *h*, are calculated prior to updating the compartments, *H*, such that compartments are maintained until all change components have been calculated. Infection in each city is dependent on the force of infection, *λ*, the product of the probability of an infectious contact resulting in transmission and the probability of a contact being infectious:

$\lambda=\beta\left(\sum H_{I}/\sum H\right)$,

where Σ*H_I_* is the total number of humans from all infectious sub-compartments and Σ*H* is divided by the total number of humans from all compartments. Newly infected humans (*E*_0_) are sampled from the susceptible population according to the force of infection:

.

Humans become infectious with a time-step-dependent probability, *p_Inc_*(*τ*), determined by the cumulative distribution of the incubation period, *F_Inc_* (described in Section 2 below):

, for $\tau=1,2,...\tau_{Inc}$.

They may also become infectious the same day they are infected,

.

Individuals recover from the infectious period following the distribution of the infectious period, *F_Inf_*, and discrete daily progression probabilities, *p_Inf_*(*τ*):

, for $\tau=1,2,...\tau_{Inf}$.

Individuals may also recover the same day they become infectious:

, for $\tau=1,2,...\tau_{Inf}$, and

.

The compartments are then updated for the next time step (*t* + 1):

$H_{S}(t+1)=H_{S}(t)-h_{S\to E_{0}}$,

$H_{E_{1}}(t+1)=h_{S\to E_{0}}-h_{S\to E_{0}\to I_{0}}$,

$H_{E_{\tau+1}}(t+1)=H_{E_{\tau}}(t)-h_{E_{\tau}\to I_{0}}$, for $\tau=1,2,...\tau_{Inc}$,

$H_{I_{1}}(t+1)=h_{S\to E_{0}\to I_{0}}-h_{S\to E_{0}\to I_{0}\to R}+\sum_{\tau=1}^{\tau_{Inc}} \left(h_{E_{\tau}\to I_{0}}-h_{E_{\tau}\to I_{0}\to R}\right)$,

$H_{I_{\tau+1}}(t+1)=H_{I_{\tau}}(t)-h_{I_{\tau}\to R}$, for $\tau=1,2,...\tau_{Inf}$,

$H_{R}(t+1)=H_{R}(t)+h_{S\to E_{0}\to I_{0}\to R}+h_{E_{0}\to I_{0}\to R}+\sum_{\tau=1}^{\tau_{Inf}} h_{I_{\tau}\to R}$,

**1.4 Vector-borne model.** The vector-borne model contains the same human compartments as the directly-transmitted model with additional compartments *V_S_*, *V_E_*, and *V_I_*, for susceptible, exposed/incubating, and infectious vectors, respectively. As for the human model, the vector incubation period, the extrinsic incubation period (EIP), is further subdivided into time-dependent sub-components.

Transitions in the human part of the model are as described above for the directly-transmitted model except for the incubation and infectious period distributions (replaced by *p_IIP_(τ)* and *p_IP_(τ)*, respectively) and the force of infection which is redefined as the vector-to-human force of infection, *λ_VH_*:

,

where *φ* is the vector density (adult female mosquitoes per person), *α* is the daily biting rate, *β_VH_* is the efficiency of vector-to-human transmission, and $V_{I}/\sum V$ is the proportion of vectors currently infectious.

New female adult mosquitoes are assumed to be completely susceptible and are generated at a rate to maintain the vector density. The number of newly emerging mosquitoes is sampled from a Poisson distribution with the mean equal to the product of the predefined vector density, *φ*, vector mortality, *µ*, and the local human population size, Σ*H*:

.

Vector mortality is implemented as the compartments are updated (below) by sampling the number of vectors surviving:

.

Susceptible vectors are exposed to the human-to-vector force of infection, *λ_HV_*, determined by the biting rate, *α*, the efficiency of human-to-vector transmission, *β_HV_*, and the proportion of humans currently infectious:

$\lambda_{HV}=\alpha\beta_{HV}\left(\sum H_{I}/\sum H\right)$,

.

Progression to the infectious state is determined by regulated by time-dependent probabilities of progression *p_EIP_*(*τ*) defined (see section 2) by the cumulative distribution function for the extrinsic incubation period, *F_EIP_*:

, for $\tau=1,2,...\tau_{EIP}$, and

.

After calculating the human and vector change components, *h* and *v*, the compartments are updated (humans as above):

,

,

, for $\tau=1,2,...\tau_{EIP}$,

.

**2. Parameterization**

**2.1 Populations.** A total of 141 cities were selected for the model. Approximate human population sizes for each city were obtained from the United Nations Statistics Division (2005 Demographic Yearbook) and Population Division (World Urbanization Prospects: The 2007 Revision Population Database). Ten cities were randomly selected to harbor vector populations. In each of these cities, the vector population size was set at a density of two susceptible vectors per person. As described above, new susceptible mosquitoes were added daily to susceptible cities at a rate equivalent to vector mortality so as to maintain a constant population size. The daily probability of vector mortality, *µ*, is set at 0.09 [4].

**2.2 Travel.** We used airline seat data (Official Airline Guide (OAG), [www.oagaviation.com/Solutions/AnalysisTools/Traffic/t100inet.html](http://www.oagaviation.com/Solutions/AnalysisTools/Traffic/t100inet.html)) augmented by sampled itinerary data for travel originating and/or ending in the United States (US Department of Transportation, [www.transtats.bts.gov/Tables.asp?DB_ID=125](http://www.transtats.bts.gov/Tables.asp?DB_ID=125)) to estimate the probability of travel between city pairs. This approach is similar to Epstein et al. [5] who used the sampled US itinerary data to fit a regression model to estimate the prevalence of travel over single, double, or multiple connections for a given city based on total seats available and the local population size. Though fitted with US data, the model was extended globally using seat data from the OAG dataset. These proportions were then used to weight a combined matrix of potential seats connecting each airport pair including all potential itineraries.

Here, we used the US sampled itinerary data to directly model the number of travelers between each city regardless of how many flights it took to make the connection. As covariates, we used characteristics of both the origin and destination city and of the potential paths between them based on network analysis. Because net travel should be roughly balanced between two cities, we only used variables that would be applicable in both directions. For example, the populations of both the origin and destination cities would be included because flow the opposite way is expected to be equivalent.

For city-specific covariates, we included the population of the city, the number of cities a given city is directly connected to (adjacency), the total number of seats departing and arriving from the city (strength), and the centrality of the city in the network (betweenness centrality) [6]. For the route between each city-pair, we included covariates for the physical distance (great circle distance for the shortest itinerary between the two cities), the minimum number of connections required, the number of alternate routes (with physical route distance within 15% of the minimum), whether or not the cities reside in the same country, the maximum possible number of seats between the two cities, and the average connectivity of airports en route (including strength, adjacency, and betweenness centrality).

The outcome was the number of itineraries between each city-pair with origin or destination in the United States. Covariates were assessed in a Poisson regression model including log-transformed covariates, polynomials, and interaction terms. Generally, the more variables included, the more accurately the model predicted the observations. However, with 2710 potential city-pairs in the model, there are ample degrees of freedom to overfit. Because of this, we focused on the covariates with strong, consistent, and non-redundant effects that described a bulk of the variability. The final model included the log population size, strength, and the total adjacency of the origin and destination cities, whether or not the cities were located in the same country, and the interaction of the country variable with each city variable.

Figure 1 shows the observed versus predicted number of itineraries for each city-pair. While the general trend is accurate and accounts for approximately 90.8% of the overall variation ($R_{\text{KL}}^{2}$[7]), the model clearly misses the mark for city-pairs with few or no sampled itineraries. This may be due to many reasons, including actual very low frequency flights or flights that were not sampled, either randomly or for unknown reasons. Unfortunately, no covariates could classify differences among these low flow city-pairs, so overestimation for travel at the low end is present in the model described here. Adjustments for zero inflation and over-dispersion provided negligible improvement and were discarded.


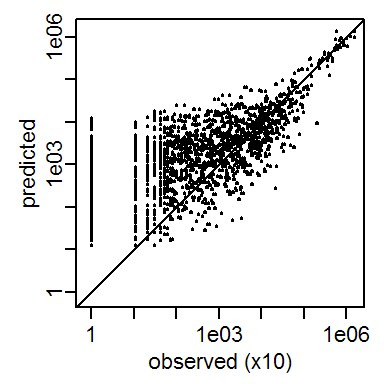


**Figure 1. Predicted versus observed travelers.** Each dot represents the predicted and observed number of travelers per year traveling between a city-pair within the model with at least one city located in the United States. The observed data are multiplied by 10 to reflect the 10% sample. The line represents perfect agreement, i.e. a 1:1 relationship. The model accounts for approximately 90.8% of the overall variation ($R_{\text{KL}}^{2}$[7]).

The duration of stay for travelers in locations other than their home city was parameterized as an exponential distribution, with mean duration of stay, *d*:

$F_{RETURN}(t)=1-e^{-t/d}$.

The mean stay was estimated to be 18 days [8]. Because the exponential hazard is independent of time, the daily probability of return, *ρ*, is equal to the cumulative distribution function over the first day:

$\rho=1-e^{-1/18}\approx0.054$.

**2.3 Infection dynamics.** The incubation period for the directly-transmitted pathogen was assumed to follow a gamma distribution with a mean of 6.37 (SD: 4.09), *F_Inc_* [9]. For the purposes of our model, we assumed that all transmission occurred during the post-disease onset, pre-hospitalization period with a mean duration of 4.85 days (SD: 3.49), also gamma-distributed, *F_Inf_* [9].

For the vector-borne model, we used log-normal distributions based on work by Nishiura and Halstead [10]. These were fitted for the intrinsic incubation period (mean: 6, SD: 1.5), and estimated based on figures for the extrinsic incubation period (mean: 11.7, SD: 0.21) and the infectious period (mean: 3.5, SD: 0.5).

The number of sub-compartments, *τ_X_*, for each distribution, *X*, was determined by ensuring that the cumulative probability of having progressed, *F_X_*(*τ*), by the last compartment would be greater than 99%. For the directly-transmitted model, *τ_Inc_* = 20 and *τ_Inf_* = 20. For the vector-borne model, *τ_EIP_* = 15, *τ_IIP_* = 15, and *τ_IP_* = 10.

Daily probabilities of progression, *p* , for each period were calculated using the relevant cumulative distribution function, *F_X_*(*τ*):

$p_{X}(0)=F_{X}(0.5)$,

$p_{X}(\tau)=\left(F_{X}(\tau+0.5)-F_{X}(\tau-0.5)\right)/\left(1-F_{X}(\tau-0.5)\right)$, for $\tau=1,2,...\tau_{X}-1$, and

$p_{X}(\tau_{X})=1$.

**2.4 Transmission parameters.** For the directly-transmitted model, we removed two important components relative to the SARS-CoV pandemic of 2002—2003: the contribution of super-spreaders to transmission and the role of hospitalization. We simplify the model here to include only average spreaders and to collapse all transmission into the pre-hospitalization period. We used the non-super-spreader *R*_0_ of 2.7 calculated by Riley et al. [1] and the average duration of the post-onset pre-hospitalization period (4.85 days [9]) to calculate an average daily infectious contact rate, *β*, of 0.56.

The vector-borne model includes three components: the rate of mosquito feeding, *α*, and the effective rates of human-to-vector transmission, *β_HV_*, and vector-to-human transmission, *β_VH_*. Field studies suggest feeding rates of 0.63—0.76 human blood meals per *Aedes aegypti* per day [11]. Here we use a mean contact rate, *α*, of 0.7. Failloux, Vazeille, and Rodhain [12] found DENV oral infection rates for *Aedes aegypti* of 13.3% to 100% with variability partly attributed to different genetic populations of the mosquito. We assume an average effective rate of human-to-vector transmission, *β_HV_*, of 0.75. The effective rate of vector-to-human transmission, *β_VH_*, is unknown and was set to 1.0 (100%) for the purpose of this study.

**3. References**

1. Riley S, Fraser C, Donnelly CA, Ghani AC, Abu-Raddad LJ, et al. (2003) Transmission dynamics of the etiological agent of SARS in Hong Kong: impact of public health interventions. Science 300: 1961-1966.

2. Colizza V, Barrat A, Barthelemy M, Vespignani A (2007) Predictability and epidemic pathways in global outbreaks of infectious diseases: the SARS case study. BMC Med 5: 34.

3. Wearing HJ, Rohani P, Keeling MJ (2005) Appropriate models for the management of infectious diseases. PLoS Medicine 2: 621-627.

4. Focks DA, Haile DG, Daniels E, Mount GA (1993) Dynamic life table model for Aedes aegypti (Diptera: Culicidae): analysis of the literature and model development. J Med Entomol 30: 1003-1017.

5. Epstein JM, Goedecke DM, Yu F, Morris RJ, Wagener DK, et al. (2007) Controlling pandemic flu: the value of international air travel restrictions. PLoS One 2: e401.

6. Barrat A, Barthelemy M, Pastor-Satorras R, Vespignani A (2004) The architecture of complex weighted networks. Proceedings of the National Academy of Sciences of the United States of America 101: 3747-3752.

7. Cameron AC, Windmeijer FAG (1997) An R-squared measure of goodness of fit for some common nonlinear regression models. Journal of Econometrics 77: 329-342.

8. U.S. Department of Commerce - International Trade Administration (2009) Profile of U.S. Resident Travelers Visiting Overseas Destinations: 2008 Outbound. Washington, D.C.: Manufacturing and Services: Office of Travel and Tourism Industries.

9. Donnelly CA, Ghani AC, Leung GM, Hedley AJ, Fraser C, et al. (2003) Epidemiological determinants of spread of causal agent of severe acute respiratory syndrome in Hong Kong. Lancet 361: 1761-1766.

10. Nishiura H, Halstead SB (2007) Natural history of dengue virus (DENV)-1 and DENV-4 infections: Reanalysis of classic studies. Journal of Infectious Diseases 195: 1007-1013.

11. Scott TW, Amerasinghe PH, Morrison AC, Lorenz LH, Clark GG, et al. (2000) Longitudinal studies of Aedes aegypti (Diptera: Culicidae) in Thailand and Puerto Rico: blood feeding frequency. Journal of Medical Entomology 37: 89-101.

12. Failloux AB, Vazeille M, Rodhain F (2002) Geographic genetic variation in populations of the dengue virus vector Aedes aegypti. Journal of Molecular Evolution 55: 653-663.
